# Microarray identifies ADAM family members as key responders to TGF-β1 in alveolar epithelial cells

**DOI:** 10.1186/1465-9921-7-114

**Published:** 2006-09-01

**Authors:** Dominic T Keating, Denise M Sadlier, Andrea Patricelli, Sinead M Smith, Dermot Walls, Jim J Egan, Peter P Doran

**Affiliations:** 1 General Clinical Research Unit, Mater Misericordiae University Hospital, School of Medicine and Medical Sciences, University College Dublin, Dublin 7, Ireland; 2 Advanced Lung Disease Programme and Lung Transplant Unit, Mater Misericordiae University Hospital; 3 School of Biotechnology, Dublin City University, Dublin, Ireland

## Abstract

The molecular mechanisms of Idiopathic Pulmonary Fibrosis (IPF) remain elusive. Transforming Growth Factor beta 1(TGF-β1) is a key effector cytokine in the development of lung fibrosis. We used microarray and computational biology strategies to identify genes whose expression is significantly altered in alveolar epithelial cells (A549) in response to TGF-β1, IL-4 and IL-13 and Epstein Barr virus.

A549 cells were exposed to 10 ng/ml TGF-β1, IL-4 and IL-13 at serial time points. Total RNA was used for hybridisation to Affymetrix Human Genome U133A microarrays. Each *in vitro *time-point was studied in duplicate and an average RMA value computed. Expression data for each time point was compared to control and a signal log ratio of 0.6 or greater taken to identify significant differential regulation. Using normalised RMA values and unsupervised Average Linkage Hierarchical Cluster Analysis, a list of 312 extracellular matrix (ECM) proteins or modulators of matrix turnover was curated via Onto-Compare and Gene-Ontology (GO) databases for baited cluster analysis of ECM associated genes.

Interrogation of the dataset using ontological classification focused cluster analysis revealed coordinate differential expression of a large cohort of extracellular matrix associated genes. Of this grouping members of the ADAM (A disintegrin and Metalloproteinase domain containing) family of genes were differentially expressed. ADAM gene expression was also identified in EBV infected A549 cells as well as IL-13 and IL-4 stimulated cells. We probed pathologenomic activities (activation and functional activity) of ADAM19 and ADAMTS9 using siRNA and collagen assays. Knockdown of these genes resulted in diminished production of collagen in A549 cells exposed to TGF-β1, suggesting a potential role for these molecules in ECM accumulation in IPF.

## Background

Idiopathic pulmonary fibrosis (IPF) is a progressive and lethal pulmonary fibrotic lung disease. It is the most common form of the idiopathic interstitial pneumonias and is unresponsive to treatment resulting in a median survival from diagnosis of 2.9 years [1]. Although the pathogenesis of IPF remains elusive, a number of conditions and risk factors are associated with the disease, including cigarette smoking, several viral proteins, and genetic predisposition to IPF [2].

During both lung development and fibrogenesis, mesenchymal signaling alters alveolar epithelial cell phenotype and regulates pneumocyte differentiation [3,4]. Effective cell function in both the epithelium and the mesenchyme is dependent on signals originating in both compartments, acting in a complimentary axis. In disease the unchecked signaling emanating from these compartments establishes persistent fibroblast migration and extracellular matrix deposition with resultant pulmonary fibrosis[5].

Injured alveolar epithelail cells release a number of profibrotic cytokines including transforming growth factors-beta-1, platelet derived growth factor tumour necrosis factor-alpha and interleukin-1 [6]. As a result of these mediators being released there a chemoattraction gradient for fibroblasts toward these areas of lung, with subsequent phenotypic differentiation.

TGF-β1 is a prominent mediator in normal wound repair without the development of fibrosis[8]. Excess production of latent TGF-β1 and active TGF-β1 has been associated with the development of temporary inflammation, however, only TGF-β1 overexpression results in fibroblast migration and proliferation with increased deposition of extracellular matrix. This suggests that inflammation in IPF is not crucial for pathogenesis but may instead be an associated phenomenon [9].

Targeted overexpression of TGF-β1 is associated with augmented fibrosis, while antagonism of the growth factor results in abrogation o the fibrotic process. TGF-β1 knockout mice die prematurely due to developmental retardation and progressive inflammation[10]; however, treatment with TGF-β1 specific antagonists in mice did not result in a significant disturbance of the immune system[11]. TGF-β1 has been shown to augment epithelial cell apoptosis and inhibition of this process has been shown to reduce fibrosis in animal models[12,13]. In other studies instillation of apoptotic cells into inflamed lungs has accelerated healing in a TGF-β1 dependent manner[14].

TGF-β1 is consistently associated with progressive fibrosis with increased expression being associated with a variety of fibrotic lung disease [15-17]. Adenovirus-mediated gene transfer of TGF-β1 resulted in severe fibrosis in animal models[15]; while αVβ6 integrin (a TGF-β1 activator) knockout mice developed lung inflammation but not fibrosis in response to bleomycin[18]. TGF-β1 displays a pivotal role in the development of a fibrotic process in animal models, however the reasons surrounding its overexpression and the predilection towards a fibrotic phenotype in this setting remains unexplained.

Interleukin (IL)-13, a Th2 cytokine, has been shown to be increased in IPF [19], while the lungs of mice injured with bleomycin display increased IL-13 and its receptor IL-13Ralpha2 [20]. The vehicle for fibrosis in response to IL-13 is activated TGF-β1 [21]. In asthmatic individuals the overexpression of IL-13 is associated with subepithelial fibrosis which has obvious implications for the development of idiopathic fibrosis [22]. IL-4 is also increased in the lungs of IPF patients and in bleomycin murine models [23]. The role for IL-4 in IPF may be two fold, limiting T-cell migration and stimulating fibrosis. IL-4 transforms fibroblasts into myofibroblasts inferring a role in the fibrotic process [24]. Through the release of TGF-β2 IL-4 initiates the release of matrix proteins by myofibroblasts while inhibition of its receptor in bleomycin-injured mice attenuates the fibrotic response [20,22].

Epstein-Barr virus (EBV) is a ubiquitous human herpesvirus associated with various diseases including infectious mononucleosis, Burkitt's lymphoma and Hodgkin's disease. A link between EBV and IPF has been suggested since Vergnon and colleagues demonstrated an elevation in the IgA levels against viral capsid antigen in IPF patients [25]. EBV usually infects the upper respiratory tract but has also been shown to infect and replicate in the lower respiratory tract. Immunohistochemical studies suggest that this replication occurs in the type II alveolar epithelial cells [26]. Further evidence for EBV involvement in IPF comes from the observation of a poorer prognosis in these patients when associated with EBV latent membrane protein 1 (LMP1) in epithelial cells [27]. LMP1 is an EBV associated protein expressed on the surface of EBV Infected cells in the latent and replicating phase [28]. Aberrant DNA found in lung tissue and serum of IPF patients suggested a mechanism by which a persistent virus can change from a latent to a productive phase via recombinatorial events [29].

In this study we explore the multifactorial nature of epithelial cell injury in pulmonary fibrosis in response to potential fibrogenic stimuli.

## Methods

### Cell culture and EBV Infection in vitro

Human alveolar epithelial cells (A549) were obtained from European Collection of Cell Cultures (Salisbury, United Kingdom) and grown *in vitro *in Hams F12 (Life Technologies, Paisley, Scotland) supplemented with 10% fetal calf serum, 146 mg/L L-glutamine, 1% penicillin and 1% streptomycin. For stimulation experiments, cells were serum starved overnight before exposure to 10 ng/ml TGF-β1, IL-4 or IL-13 (Sigma) for the indicated time points, control samples were maintained in serum free conditions.

To effect virus infection, A549 cells were co-cultured with Akata cells (an Epstein-Barr virus-negative cell line infected with recombinant EBV carrying the neomycin resistance gene) [30]. The Akata cell line was maintained in RPMI 1640 (Life Technologies, Paisley, Scotland) supplemented with 10% FBS, 2 mM L-glutamine, 100 U/ml Penicillin, 100 μg/ml streptomycin and 700 μg/ml G418. The viral lytic cycle was induced in the Akata cells by adding goat anti-human serum Immunoglobulin G (Sigma) at 100 μg/ml. After four hours the Akata cells were added to the A549 wells at a concentration of 5 × 10^5^/ml. After two days incubation half the medium was replaced with medium containing 5% FCS. Following a further 4 days incubation the media containing the Akata cells was removed, pelleted down and resuspended in M5 media (Calcium free DMEM supplemented with 5% Horse Serum, 2 mM glutamine, cortisol, 2 ng/ml EGF, 10 mg/ml Insulin, 100 ng/ml cholera toxin, 100 U/ml Penicillin and 100 μg/ml streptomycin). 2 mls of this cell suspension was then placed onto the wells and left to incubate for 2 days. The wells were washed with PBS and the media replaced with fresh medium containing 10% FCS. Following incubation for 24 hours 700 ng/ml G418 (Sigma) was added to select for EBV-infected A549 cells.

### Western blot analysis

Latent infection of the A549 cells with EBV was confirmed by the detection of latent membrane protein 1 (LMP1) by Western blot. After removal of EBV/A549 cells from flasks protein lysates were prepared by boiling for 10 minutes in 2% SDS, 100 mM NaCl, 0.01 M Tris-HCl, 5% β-Mercaptoethanol, 1 mM EDTA, 100 μg of phenylmethylsulfonyl fluoride/ml, and 2 μg of leupeptin/ml. The product was then sonicated on ice and clarified at room temperature by centrifugation at 12,000 rpm for 10 min. Protein fractionated by discontinuous SDS-5–10% polyacrylamide gel electrophoresis and blotted onto a nitrocellulose filter. Anti-LMP1 CS1-4 antibody (University of Wales, Cardiff) cocktail diluted to 1:100 in Blotto (5% skim milk and 0.1% Tween 20 in Tris-buffered saline) was used to probe the filters at 4°C overnight. Alkaline phosphatase-conjugated sheep anti-mouse immunoglobulin G (IgG) (Promega) was used to detect immunocomplexes, which were visualized using 5-bromo-4-chloro-3-indolylphosphate (BCIP)-nitroblue tetrazolium liquid substrate (Sigma).

### RNA extraction and gene array analysis

Following stimulation of A549 cells with 10 ng/ml TGF-B1 for 15 mins, 30 mins, 2 hour and 4 hours, RNA isolation, cDNA synthesis, *in vitro *transcription and microarray analysis were performed as previously reported [31] and in accordance with Affymetrix protocols(Affymetrix, Santa Clara, California). Arrays were scanned with a confocal scanner (Affymetrix). Each RNA sample derived from an individual well and 15 min, 30 min, 2 hour and 4 hour *in vitro *exposures were microarrayed in duplicate on HU133 Affymetrix chips. Image files were obtained through Affymetrix GeneChip software (MAS5) and subsequently robust multichip analysis (RMA) was performed. To ensure the average was statistically significant a t-test and p-value were generated. Only those genes with a p-value ≤ 0.01 were included in subsequent bioinformatic analysis. Expression data was further probed to identify those genes whose expression is altered. Expression data for each time point was compared to control and a signal log ratio of 0.6 or greater (equivalent to a fold change in expression of 1.5 or greater) was taken to identify significant differential regulation. Using normalised RMA values, unsupervised average linkage hierarchical cluster analysis was performed using an Eisen software program [32]. Cluster analysis is a group of mathematical techniques for the identification of patterns in large datasets. Briefly, a distance metric is used to calculate the similarity between the expression profiles of a group of genes. The more similar the expression profiles of genes are, the closer they are placed together on a dendrogram or tree. A list of 312 extracellular matrix proteins or modulators of matrix turnover was curated via the publicly available Onto-Compare and Gene-Ontology databases [33].

### Real-time PCR

Reverse transcription was carried out using the Promega reverse transcription system. 1 μl of Oligo(dT)_15 _(0.5 μg/μl) was mixed with 1 μg of total RNA and the volume brought to 5 ml with sterile nuclease-free water. The mixture was incubated at 70°C for 10 min and then placed on ice. Once cooled the following was added for a 20 μl reaction: 2 μl 10× transcription buffer, 0.5 μl RNasin (40 units/μl), 2 μl dNTP mix (10 mM), 4 μl MgCl_2 _(25 mM), 1 μl AVM-RT (Avian Myeloblastic Virus Reverse Transcriptase)(20 units/μl), and brought to a final volume of 20 μl with nuclease free water. The sample was mixed by repeated pipetting and then centrifuged to collect the sample at the bottom of the PCR reaction tube. The mixture was incubated at 37°C for an hour and heated to 95°C for 2 min in order to inactivate the enzyme. The subsequent cDNA was stored at 4°C until required.

Real time RT-PCR was performed on a TaqMan ABI 7700 Sequence Detection System^® ^(AppliedBiosystems, Weiterstadt, Germany) using heat activated AmpliTaq Gold, DNA polymerase (Amplitaq Gold, Applied Biosystems, Weiterstadt, Germany) as previously described [34]. The ribosomal 18S was used as an endogenous control for normalisation of the target genes. Its primer and probe were supplied as a PDAR (predeveloped assay reagent) from Applied Biosystems with the probe labelled with VIC at the 5' end. Primer and probes for the genes of interest were designed in PrimerExpress^® ^version 2.0(AppliedBiosystems). The probes for the target genes were labelled with fluorescent dye, FAM on the 5' end and the quencher TAMRA onthe 3' end. PCR reactions were set up in separate tubes with TaqMan Universal PCR Master Mix from Applied Biosystems. Optimal concentration of primers and probes were 200 nM for probe, 300 nM for its primers, and 100 nM reaction mix for PDARs. cDNA was amplified on the 7700HT detection system (Applied bioscience) at default thermal conditions: 2 min @50°C, 10 min @95°C for enzyme activation and the 40 cycles of 15 sec @95°C for denaturation and 1 min @60°C for annealing and extension. Controls consisting of distilled H_2_O were negative in all runs. All measurements were performed in triplicate for each time point.

Following cycling, to ensure specificity, melt curve analysis was carried out to verify the amplification of PCR products starting at 65°C and ramping to 90°C at .1°C/sec. One peak in the melt curve indicated no secondary, non-specific products were formed. All results were compared to those for unstimulated A549 cells and analysed using the delta delta Ct method. All experiments were performed in triplicate for each time point.

### Gene silencing by RNA interference

Knock down of gene expression was achieved using RNA interference. Two siRNA duplexes were designed and synthesised for silencing ADAM19 and ADAMTS9. (Qiagen Inc. CA, USA). A chemically synthesized non-silencing siRNA duplex that had no known homology with mammalian genes was used to control for non-specific silencing events. 2 × 10^5 ^A549 cells were added to each well of a 6-well plate in 3 ml growth media and incubated under the standard conditions of 37°C and 5 % CO_2 _in a humid incubator for 24 hr. A sufficient amount of growth medium was added to 5 μg siRNA and 30 μl RNAifect (Qiagen) to bring the final volume to 100 μl. Following incubation, media was removed from the cells and this mix was added drop-wise. 3 ml growth medium was added and the cells were incubated for 48 hr under standard conditions. Following this, all growth media was removed and cells were washed with sterile PBS. 1 ml TRIzol™ (Sigma) was added to each well and left for 10 min at room temperature with occasional shaking. 200 μl chloroform was added and the mixture was shaken, left at room temperature for 15 min and centrifuged at 13,000 g at 4°C for 15 min. The upper aqueous layer was transferred to a fresh 1.5 ml tube. 0.5 ml ice-cold isopropanol was added to the aqueous phase, shaken and left to stand on ice for 10 min before it was centrifuged at 13000 g at 4°C for 10 min. The supernatant was removed and 1 ml of sterile 75 % ethanol was added to wash the pellet by gentle vortexing and centrifugation at 7500 g for 5 min. The ethanol was removed and the pellet was allowed to air-dry for 5 min. Pellets were resuspended in 50 μl 0.1 % DEPC treated H_2_O by heating at 60°C for 15 min. All RNA was stored at -80°C.

### Collagen Assay

Sircol collagen assay (Biocolour) was performed as per manufacturer's guidelines. The dye reagent contains sirius red in picric acid. Sirius Red is an anionic dye with sulphonic acid side chain groups. These groups react with the side chain groups of the basic amino acids present in collagen under specific conditions permitting determination of mammalian collagens types I to V. Briefly 100 μl aliquots of cell culture supernatant were incubated with the dye reagent by gentle mixing for 30 mins at room temperature. The dye-bound collagen was pelleted by centrifugation at 10000 g for 10 mins. Unbound dye was removed by aspiration of the supernatant following centrifugation. The collagen dye complex was washed with 500 μl ethanol to ensure complete removal of unbound dye. The collagen bound dye pellet was recovered by solubilization in an alkaline solution. Absorbance of bound collagen at 540 nm was determined using a spectrophotometer. Bound collagen concentration was determined by comparison with absorbance standard curve of known concentration samples.

## Results

### Global changes in gene expression in response to TGF-β1

**Figure 1:**
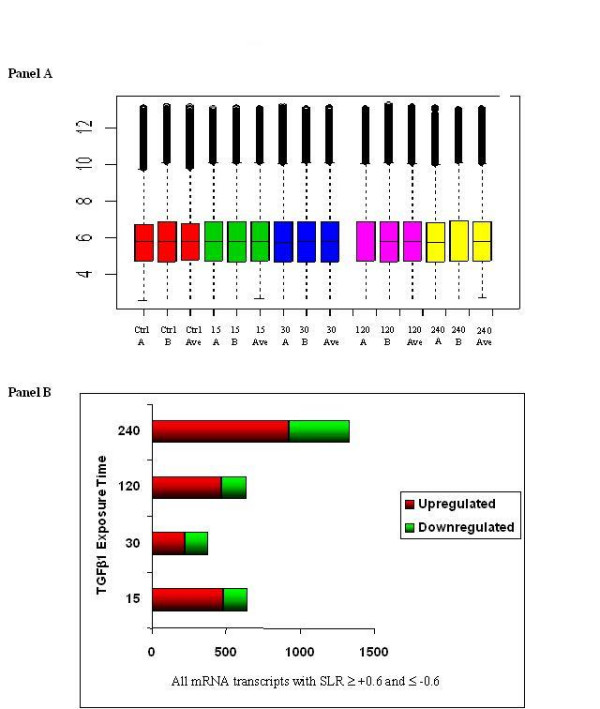
**Global changes in alveolar epithelial cell gene expression following exposure to 10 ng/ml TGF-β1**. Panel A shows a boxplot of normalised data and computed average arrays for each time point demonstrating comparability of the normalised data. Each array is performed in duplicate (A and B) and is shown beside their computed average (Ave). Arrays were performed for control (Ctrl), and TGF-β1 stimulation at 15, 30,120, and 240 minutes. Panel B shows a summary of the observed alterations in gene expression at all the time points following TGF-β1 stimulation. Genes were defined as upregulated when signal log ratio (SLR) >0.6 and downregulated when SLR<-0.6.

Exposure of A549 alveolar epithelial cells to 10 ng/ml TGF-β1 was associated with significant changes in gene expression. For all time points data was normalised using RMA express and an average expression measure for each time point used to identify alterations in gene expression. RMA normalised data was found to be comparable across the time series with the computed average expression aligning to the individual chip hybridisation boxplots (Figure 1, Panel A). Distinct temporal patterns of gene expression were observed throughout the time course exposure, with significant altered expression following 15 minutes exposure. The total number of genes altered was lower following 30 minutes with a sustained increase seen over the remaining time points. The same temporal pattern of gene expression alterations was observed for both up and down regulated transcripts. Of the 22,216 gene sequences represented on the Affymetrix HGU133A oligonucleotide microarray 2.9% (649) genes), 1.7% (383 genes), 2.89% (643 genes) and 6.01% (1339 genes) were significantly altered following 15 minutes, 30 minutes, 2 hour and 4 hour exposure to TGF-β1 respectively (Figure 1, Panel B). Tables 1 and 2 highlight the genes whose mRNA levels were most strikingly altered at 15, 30, 120 and 240 minutes post TGF-β1 exposure.

**Table 1:** Genes undergoing most striking up-regulation at 15, 30, 120 and 240 minutes post TGF-β1 exposure.

**Accession**	**Gene**	**A549 Vs. 15 mins**
NM_001554	Cysteine-rich, angiogenic inducer, 61	1.1
NM_006745	Sterol-C4-methyl oxidase-like	1.0
NM_004735	Leucine rich repeat interacting protein 1	0.9
AB023420	Heat shock 70KDa protein 4	0.8
NM_018063	Helicase, lymphoid-specific	0.8
NM_006621	S-adenosylhomocysteine hydrolase-like 1	0.8
NM_006364	Sec23 homolog A (S. cerevisiae)	0.7
		
**Accession**	**Gene**	**A549 Vs. 30 mins**

NM_003670	Basic helix-loop-helix domain containing, class B, 2	1.6
NM_002165	Inhibitor of DNA binding 1	1.6
NM_002229	Jun B proto-oncogene	1.6
NM_005655	TGFB inducible early growth factor	1.4
NM_001554	Cysteine-rich, angiogenic inducer, 61	1.2
NM_002228	v-jun sarcoma virus 17 oncogene homolog	1.2
NM_004907	Immediate early protein	1.1
		
**Accession**	**Gene**	**A549 Vs. 120 mins**

NM_000602	Serine proteinase inhibitor, clade E	3.0
S69738	Chemokine ligand 2	2.8
AL574210	Serine proteinase inhibitor, clade E	2.6
NM_002229	Jun B proto-oncogene	2.5
NM_004428	Ephrin-A1	2.3
NM_000641	Interleukin 11	2.2
NM_003897	Immediate early response 3	2.2
		
**Accession**	**Gene**	**A549 Vs. 240 mins**

NM_000602	Serine proteinase inhibitor, clade E	3.2
AL574210	Serine proteinase inhibitor, clade E	2.7
NM_001901	Connective tissue growth factor	2.7
S69738	Chemokine ligand 2	2.7
NM_000641	Interleukin 11	2.3
NM_003897	Immediate early response 3	2.0
NM_016109	Angiopoietin-like 4	1.9

**Table 2:** Genes undergoing most striking down-regulation at 15, 30, 120 and 240 minutes post TGF-β1 exposure.

**Accession**	**Gene**	**A549 Vs. 15 mins**
X7230	Corticotropin releasing hormone receptor	-0.5
NM_014012	RAS-like GTP-binding	-0.5
NM_018028	Hypothetical protein FLJ10211	-0.5
AW003030	Splicing factor 3b, subunit 1	-0.5
XM373433	Hypothetical protein BC002926	-0.5
NM_015638	Transient receptor potential cation channel	-0.5
NM_020660	Connexin-36	-0.5
		
**Accession**	**Gene**	**A549 Vs. 30 mins**

NM_005469	Peroxisomal acyl-CoA thioesterase	-0.5
AL571723	SPRY domain-containing SOCS box protein SSB-3	-0.5
N39314	Mitochondrial carrier triple repeat 1	-0.5
X02761	Fibronectin 1	-0.5
NM_022829	Solute carrier family 13	-0.5
AK026737	Fibronectin 1	-0.5
NM_020660	Connexin-36	-0.5
		
**Accession**	**Gene**	**A549 Vs. 120 mins**

AB012305	Cyclin-dependent kinase 2	-0.5
NM_001086	Arylacetamide deacetylase	-0.5
NM_004083	Methionine-tRNA synthetase	-0.5
NM_006933	Mitochondrial ribosomal protein S6	-0.5
NM_013453	Sperm protein associated with the nucleus, X-linked	-0.5
NM_020660	Connexin-36	-0.5
AK026737	Fibronectin 1	-0.5
		
**Accession**	**Gene**	**A549 Vs. 240 mins**

NM_005345	Heat shock 70Kda protein 1A	-0.5
NM_014158	Core 1 UDP-galactose	-0.5
NM_005203	Collagen, type XIII, alpha 1	-0.5
L03203	Peripheral myelin protein 22	-0.5
NM_001417	Eukaryotic translation initiation factor 4B	-0.5
NM_017960	Hypothetical protein FLJ20808	-0.5
AB012305	Cyclin-dependent kinase 2	-0.5

### Baited Cluster analysis Identifies Extracellular Matrix Associated Genes as major responders to TGF-β1 exposure

**Figure 2:**
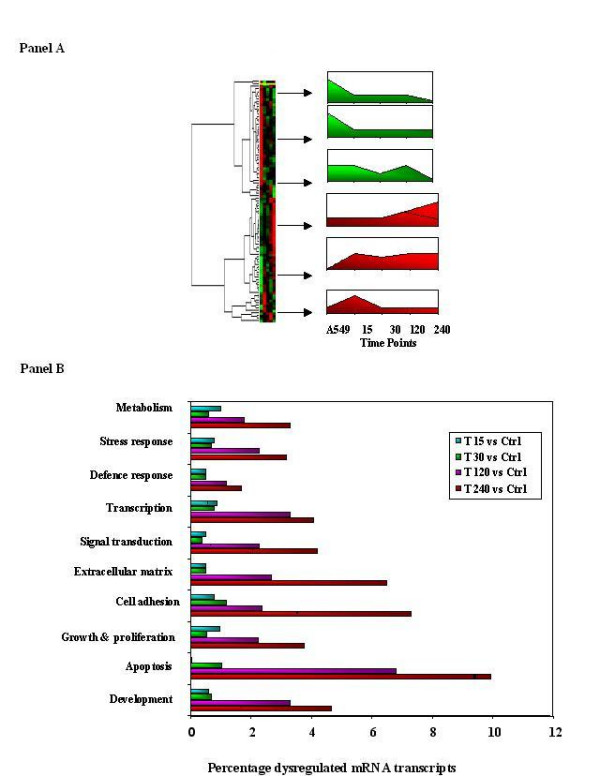
**Functional classification of global gene expression changes in alveolar epithelial cells elicited by TGF-β1**. Panel A shows a cluster dendrogram of all arrays demonstrating aggregation of the data representative of each time point. Array image files were used as input to RMAExpress for normalization. In panel B all significantly dysregulated genes (SLR < -0.6 & SLR > 0.6) were used to classify the TGF-β1 induced transcriptome in terms of biological function of the perturbed genes. Shown is a bar chart describing the percentage of dysregulated transcripts, from each family found to be significantly changed at each time point.

Figure 2, panel A shows the result of unsupervised hierarchical cluster analysis of all alveolar epithelial cell genes whose expression is significantly altered in response to TGF-β1. As can be seen groups of genes are found to cluster together depending on the kinetics of their altered expression. Having delineated the global transcriptomic response of alveolar epithelial cells to TGF-β1, we categorized the significantly perturbed genes according to their biological function. This approach permits rapid annotation of large datasets for the identification of functional patterns of dysregulation. All significantly perturbed genes were used as input in classification searches. Figure 2 Panel B shows the overall pattern of regulation of key functional families throughout the time course exposure. All gene families studied were found to increase over time, reflecting the increased transcriptomic activity in the latter time points.

**Figure 3:**
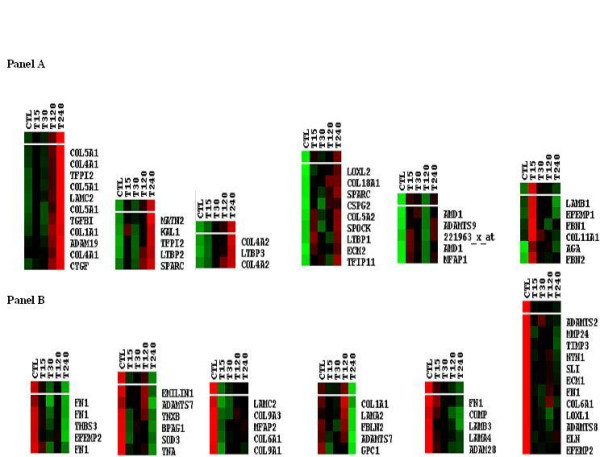
**Extracellular matrix family gene expression in response to TGF-β1**. A list of 312 extracellular matrix associated genes obtained from Onto-Compare was used to scan for genes undergoing significant perturbation in TGF-β1 stimulated cells. Panel A and B illustrate ECM genes whose expression was found to increase and decrease respectively. Arrays shown in this figure are control (Ctl), TGF-β1 stimulated time points 15 min (T15), 30 min (T30), 120 min (T120), and 240 min (T240).

Of the 312 ECM genes displayed on the microarray 95 were significantly altered in this setting. Figure 3 illustrates the extracellular matrix associated genes whose expression was altered in response to TGF-β1 including matrix proteins, such as members of the collagen family and growth factors known to be involved in matrix regulation, including connective tissue growth factor and transforming growth factor. Figure 3, Panel A and B show the expression patterns of up and downregulated transcripts respectively.

### TGF-β1 stimulation drives ADAM family gene expression in alveolar epithelial cells

Of note with respect to the mechanisms of fibrotic lung injury was the finding of coordinate differential regulation of ADAM gene family members. We focused on four ADAM family members identified in the ECM cluster of our oligonucleotide microarrays. ADAM19 and ADAMTS9 were increased in response to TGF-β1 exposure, whilst mRNA levels of ADAM28 and ADAMTS8 were reduced.

**Figure 4:**
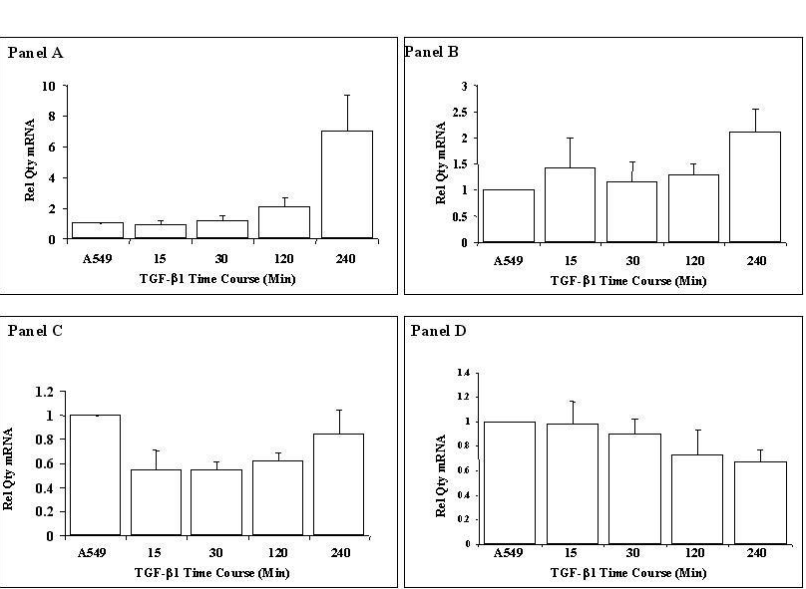
**ADAM mRNA expression levels in TGF-β1 stimulated alveolar epithelial cells**. Confirmation of the oligonucleotide microarray identified ADAM 19, ADAMTS9, ADAMTS8 and ADAM 28 (Panels A, B, C and D respectively.) by quantitative real time PCR. All expression values were normalised to GAPDH to control for equivalence of loading. Data are quoted relative to control, which has a value of 1.

Microarray findings were validated using quantitative real time PCR. ADAM19 expression was significantly enhanced by 6 fold at 4 hours (figure 4, panel A). ADAMTS9 analysis showed an increase in response to TGF-β1 exposure after 15 minutes and reaching a significantly elevated level of 2 fold at 4 hours (figure 4, panel B).

ADAMTS8 was identified as being downregulated in response to TGF-β1. Real time quantitative PCR confirmed the TGF-β1 responsiveness of this gene in alveolar epithelial cells at all but the 4 hour exposure time points, (Figure 4, Panel C). Downregulation of ADAM 28 mRNA was confirmed by quantitative real-time PCR at 4 hours (Figure 4, Panel D).

These data confirm the microarray-identified alterations in ADAM family members in alveolar epithelial cells in response to TGF-β1.

### mRNA levels of ADAM Family members are altered in response to endogenous and exogenous stimuli

Having determined that exposure of alveolar epithelial cells to TGF-β1 resulted in coordinate regulation of ADAM family members we explored the effect of other fibrotic stimuli on ADAM expression particularly IL-13 and IL-4.

**Figure 5:**
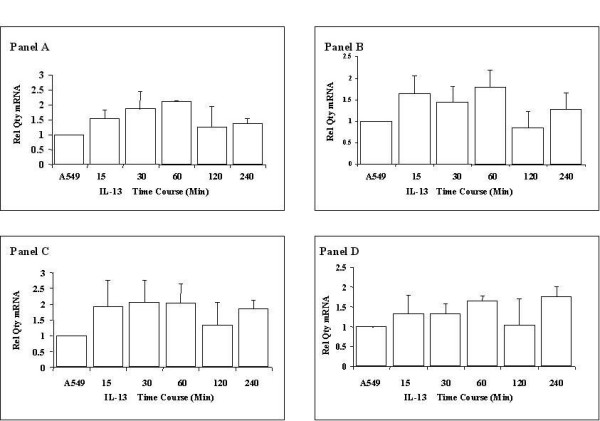
**ADAM mRNA expression in response to interleukin-13**. A549 alveolar epithelial cells were exposed to 10 ng/ml Interleukin 13; mRNA expression of ADAM 19 (Panel A), ADAM 28 (Panel B), ADAMTS9 (Panel C) and ADAMTS8 (Panel D) quantified by Real Time PCR is shown. All expression values were normalised to GAPDH to control for equivalence of loading. Data are quoted relative to control, which has a value of 1.

Alveolar epithelial cells were exposed to 10 ng/ml IL-13 for 15, 30, 60, 120 and 240 mins and ADAM gene expression assessed by quantitative Real time PCR. Figure 5 Panel A and B demonstrates the induction of the TGF-β1 upregulated genes, ADAM19 and ADAMTS9 in response to IL-13 stimulation. ADAM 19 was found to be significantly induced at the 60 min time point post IL-13 exposure, then the levels returning to almost baseline by 240 min. ADAMTS9 was significantly enhanced at all time points, with maximal induction seen in the 240 min setting. In contrast IL-13 was found to have little effect on the TGF-β1 downregulated genes, ADAM28 and ADAMTS 8 (Figure 5, Panel C and D).

**Figure 6:**
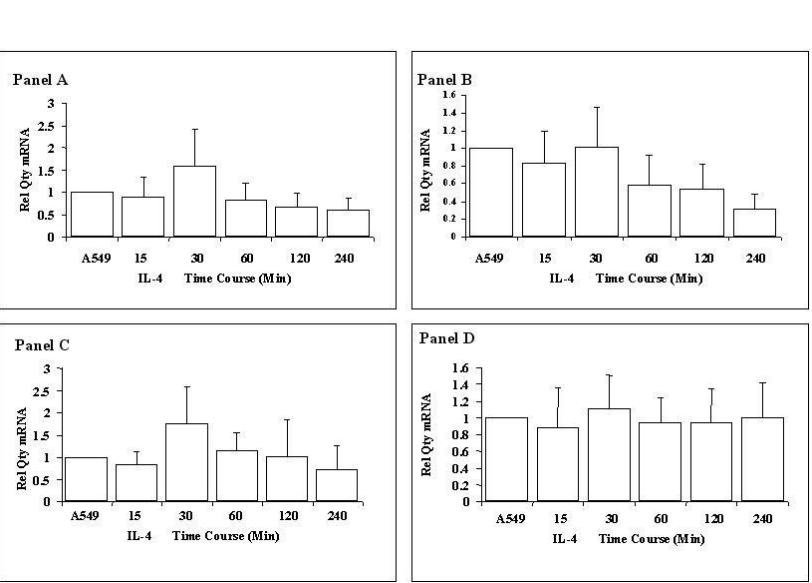
**ADAM mRNA expression in response to interleukin-4**. A549 alveolar epithelial cells were exposed to 10 ng/ml Interleukin 4. Shown are mRNA expressions of ADAM 19 (Panel A), ADAM 28 (Panel B), ADAMTS9 (Panel C) and ADAMTS8 (Panel D) quantified by Real Time PCR. All expression values were normalised to GAPDH to control for equivalence of loading. All measurements were completed in triplicate. Data are quoted relative to control, which has a value of 1.

Interleukin 4 exposure had no significant effect on the expression of ADAM 19 or ADAMTS9. There was a general trend towards downregulation of these transcripts, the opposite effects to that seen with TGF-β1. Suppression of the TGF-β1 downregulated genes ADAM 28 and ADAMTS8 by IL-4 was interrogated by real time PCR. However only the latter time points of IL-4 exposure produced a statistically significant change in ADAM 28 expression (Figure 6, panel C).

**Figure 7:**
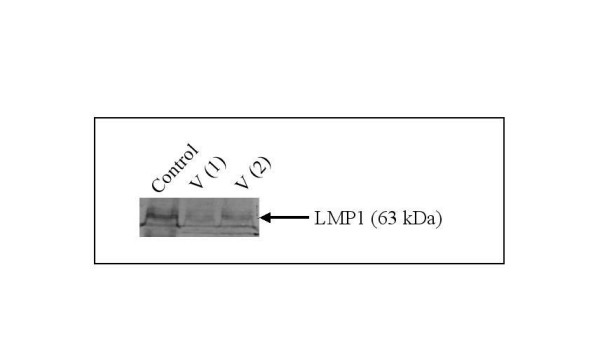
**Epstein Barr Virus infection of alveolar epithelial cells**. EBV infection of A549 alveolar epithelial cells was confirmed by western blotting for the viral protein LMP1. LMP1 was detected in Akata cells (Control) as a positive control. Lanes V1 and V2 demonstrate LMP-1 protein expression in infected A549 alveolar epithelial cells. Each lane represents protein taken from separate experiments containing 4 × 10^5 ^cells.

**Figure 8:**
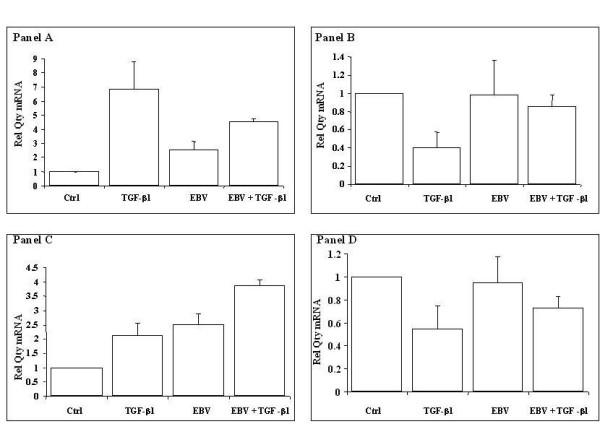
**ADAM mRNA expression levels in Epstein Barr Virus infected alveolar epithelial cells**. Using real time PCR the effect of EBV infection and TGF-β1 costimulation on ADAM expression was determined. ADAM expression in A549 cells (C), A549 cells stimulated with TGF-β1 (TGF), EBV infected A549 cells with (VTGF) and without (V) TGF-β1 are shown. Expression levels of ADAM 19 (Panel A), ADAM 28 (Panel B), ADAMTS9 (Panel C) and ADAMTS8 (Panel D) are shown. All expression values were normalised to 18S rRNA to control for equivalence of loading. All measurements were completed in triplicate. Data are quoted relative to control, which has a value of 1.

EBV infection of A549 cells was confirmed by western blot expression of latent membrane protein 1 (LMP1) in A549 infected cells (Figure 7). Having confirmed the viral infection of A549 cells with EBV we determined the effect of this infection on ADAM gene expression. ADAM19 and ADAMTS9 were found to be significantly induced in virus infected alveolar epithelial cells. Stimulation of these infected cells with TGF-β1 resulted in further enhanced expression of these genes, suggesting a synergistic activity of EBV and TGF-β1 in the fibrotic lung (Figure 8, panel A and B). Of note was the finding that EBV infection had no statistically significant effect on ADAM 28 gene expression either alone or in conjunction with TGF-β1. Decreased expression of ADAMTS8 was found in virus infected TGF-β1 exposed alveolar epithelial cells (Figure 8, Panel C and D).

### ADAM 19 and ADAMTS9 Gene Silencing inhibits lung fibrosis in vitro

**Figure 9:**
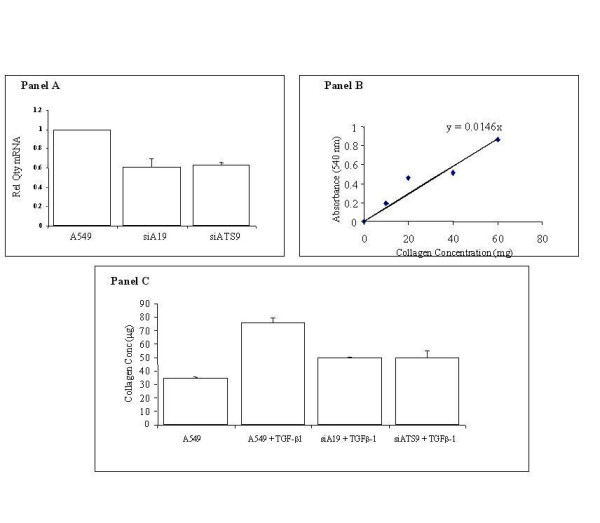
**ADAM 19 and ADAMTS9 gene knockdown abrogate TGF-β1 induced collaged production**. Successful knockdown of ADAM19 and ADAMTS9 mRNA levels are shown in panel A using real time PCR. In panel B a standard curve for quantification is shown along with a bar chart illustrating reduced collagen deposition in response to TGF-β1 in ADAM19 and ADAMTS9 knockdown alveolar epithelial cells, respectively. All measurements were completed in triplicate.

To determine the biological importance of enhanced ADAM 19 and ADAMTS9 expression in response to TGF-β1 exposure we evaluated the effect of gene knock down on the cellular phenotype. To achieve this goal, specific small interfering RNA oligonucleotide [35] probes were designed and transfected into A549 alveolar epithelial cells using the lipofectamine strategy as described. Following transfection knockdown of the genes was confirmed by quantitative PCR (Figure 9, Panel A). Transfected cells were exposed to 10 ng/ml TGF-β1 for four hours as previously described and collagen (Types I-V) deposition, as a hallmark of fibrosis, was determined using the Sircol assay kit. Figure 9 panel B demonstrates reduced collagen deposition in both ADAM 19 and ADAMTS9 siRNA transfected cells. These data suggest that the upregulation of ADAM 19 and ADAMTS9 by TGF-β1 in the setting of lung fibrosis plays a role in the deposition of collagen in the cellular milieu, thereby contributing to extracellular matrix deposition and lung scarring.

## Discussion

To delineate the molecular events in the pathogenesis of pulmonary fibrosis we utilised an oligonucleotide microarray strategy to identify alveolar epithelial genes and gene clusters whose expression is altered in response to TGF-β1. These studies identified a large number of genes whose expression was altered in a temporal fashion. Functional classification of the transcriptomic response identified coordinate expression of important functional groups of genes in response to TGF-β1. Particular focus was made on genes that are associated with extracellular matrix and it's remodelling. We curated the Affymetrix Human Genome HU133a microarray to obtain a list of such genes represented on the chip. Approximately 30% of this gene cohort was found to be differentially regulated further underpinning the relative contribution of matrix molecules and mediators to the response to TGF-β1. Representative among these extracellular matrix genes were growth factors, collagens and members of the ADAM gene family. The interaction of epithelial cells with other local and infiltrating cell types drives the further articulation of the fibrotic response in the lung. The ability of ADAM family members to regulate the interaction of these cell types and indeed to regulate their interaction with the extracellular matrix raises the hypothesis that these mediators are important in driving the local fibrotic milieu in the lung.

The ADAM gene family encompasses the ADAM (A disintegrin and metalloproteinase domain containing protein) and ADAMTS (A disintegrin-like and metalloproteinase with thrombospondin type I motif) proteins. The ADAMTSs are soluble proteins that can bind to the ECM through the TS motifs. To date 34 members of the ADAMs family have been described, of which approximately 50% have been demonstrated to have protease activity. The substrates of ADAM protease activity are either integral membrane proteins or extracellular matrix proteins. In addition to their proteolytic activity, a number of ADAMs have been shown to bind integrins, via their disintegrin domain [36]. This dual activity of ADAMs suggests that these molecules are key mediators of biological processes such as cell-cell adhesion, ectodomain shedding, myoblast fusion and development. In the context of lung disease, this family is noteworthy. The possible role for ADAM family genes in lung fibrosis has been intimated by the observation that ADAM 10 autoantibody is associated with dermatomyositis related lung fibrosis and was shown to be present in one patient with IPF [37]. ADAM33 polymorphisms have also been shown to play a role in the development of asthma and also in disease progression via effects on airway remodelling [38].

The ADAM gene family is characterised by members that possess both proteolytic and cell-cell and cell-matrix interaction promoting activities. Thus the matrix proteolytic activities of ADAMs may represent an important facet of the development of pulmonary fibrosis, as their differential expression may alter the balance of matrix turnover. ADAM genes identified in this study have previously been associated with ventricular septal defects and valvular defects (ADAM19) [39], fibrotic eye disease (ADAMTS9) [40], disrupting angiogenesis (ADAMTS8) [41], and cell adhesion (ADAM 28) [42]. Functional assessment of the role of these genes in the setting of the lung was described using a gene knock down based strategy. These investigations showed an effect on collagen deposition in cells where the ADAM gene was knocked down, thus illustrating the importance of dysregulation of this gene family in the setting of lung fibrosis.

Having demonstrated the responsiveness and functional activity of ADAM family members in response to TGF-β1 we explored their induction in response to other endogenous and exogenous pro-fibrotic insults. We determined the effect of IL-4 and IL-13 on alveolar epithelial cell ADAM production. Whilst the specific dysregulation of ADAM family members by these cytokines was different to that elicited by TGF-β1, the general trend of ADAM response remained the same. These data suggest that the ADAM family response may be more indicative of the overall fibrotic activity rather than being a stimulus specific effect. These findings lend further weight to the argument that ADAM genes are central in mediating fibrosis as opposed to being an artefact of the experimental model.

Of particular note was the finding that viral infection itself resulted in ADAM family dysregulation in these cells, an effect that was further enhanced in experiments where repeated injury consisting of both EBV infection and TGF-β1 exposure induced ADAM gene expression. Whilst the ADAM gene expression was not as great in EBV infected cells as it was in A549 cells stimulated with TGF-β1, a significant rise in ADAM19 and ADAMTS9 expression was observed. The combined stimulation of A549 cells with both EBV and TGF-β1 resulted in an additional increase in ADAM19 and ADAMTS9 expression.

These data strengthen the hypothesis that the initiation and progression of lung fibrosis is due to injury of the epithelial cell with abnormal healing. Repetitive injury due to EBV infection is one plausible mediator for the development of fibrosis. It is of note that the ADAM response was consistently observed following different experimental exposures, underpinning the fact that these observed changes in ADAM expression and functionality may be of clinical importance and are not merely representative of experimental and model derived artefacts.

The data highlight the complex mechanism underpinning the initiation and progression of IPF and underline mechanisms for both the multifactorial nature of disease initiation and the mediators of deposition of extracellular matrix.
